# P-1735. Species-Specific Survival Analysis of Candidemia in Costa Rica: Prognostic Factors and Clinical Outcomes (2007-2023)

**DOI:** 10.1093/ofid/ofaf695.1906

**Published:** 2026-01-11

**Authors:** Juan Villalobos Vindas, Jose A Castro Cordero, Elvira Segura Retana, Heylin Estrada Murillo, Alvaro A Aviles Montoya, Carlos Ramírez Valverde, Saúl Quirós Cárdenas, Laura Villalobos González

**Affiliations:** Caja Costarricense de Seguro Social, San José, San Jose, Costa Rica; Caja Costarricense de Seguro Social, San José, San Jose, Costa Rica; Caja Costarricense de Seguro Social, San José, San Jose, Costa Rica; Caja Costarricense del Seguro Social, La Unión, Cartago, Costa Rica; Caja Costarricense de Seguro Social, San José, San Jose, Costa Rica; Caja Costarricense del Seguro Social, La Unión, Cartago, Costa Rica; CCSS, San Jose, San Jose, Costa Rica; Caja Costarricense de Seguro Social, San José, San Jose, Costa Rica

## Abstract

**Background:**

Candidemia is associated with high mortality rates, but limited data exist on species-specific survival outcomes in Latin America. This study analyzes candidemia survival patterns in Costa Rica over 17 years.

**Figure:**
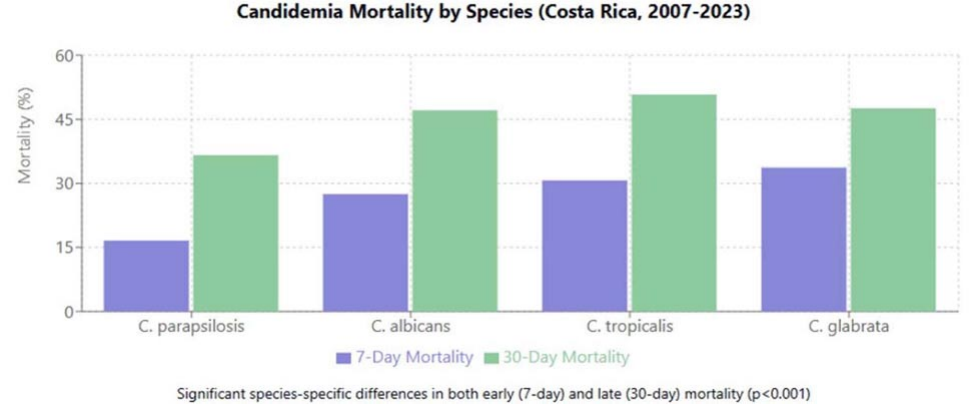
Candidemia Mortality by Species (Costa Rica, 2007-2023) Candidemia Mortality by Species (Costa Rica, 2007-2023)

**Figure:**
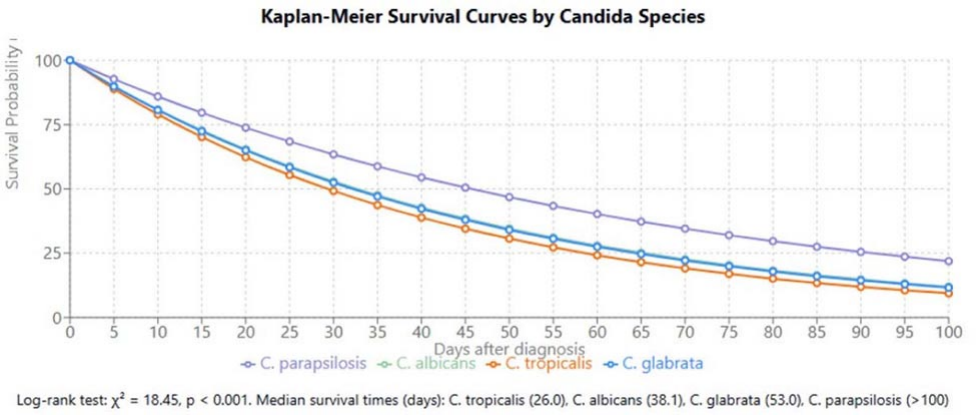
Kaplan-Meier Survival Curves by Candida Species Kaplan-Meier Survival Curves by Candida Species

**Methods:**

We conducted survival analysis on 2,128 candidemia cases from two tertiary hospitals (2007-2023), examining mortality rates at 7 and 30 days, and using Kaplan-Meier analysis to determine species-specific survival differences.

**Figure:**
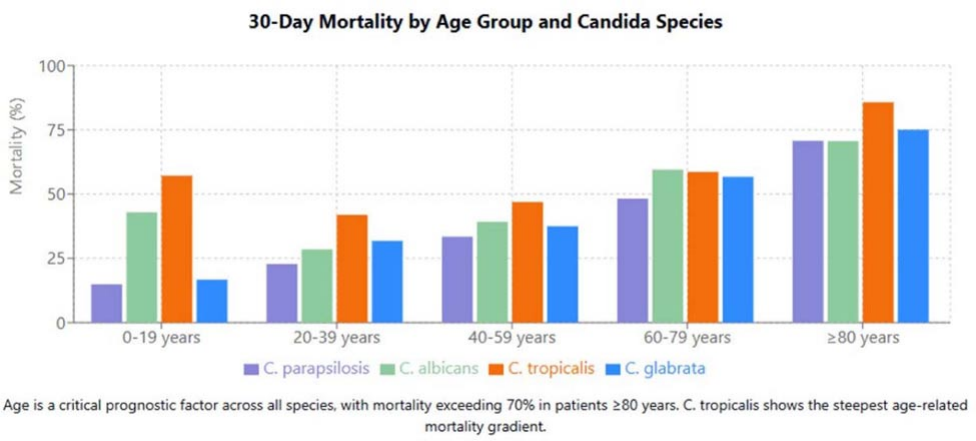
30-Day Mortality by Age Group and Candida Species 30-Day Mortality by Age Group and Candida Species

**Results:**

Early mortality (7 days) differed significantly by species (χ²=47.02, p< 0.001): C. parapsilosis 16.6%, C. albicans 27.5%, C. tropicalis 30.7%, and C. glabrata 33.7%. Thirty-day mortality maintained this pattern (χ²=26.50, p< 0.001): C. parapsilosis 36.6%, C. albicans 47.1%, C. tropicalis 50.8%, and C. glabrata 47.6%. Kaplan-Meier analysis confirmed significant survival differences (Log-rank: χ²=18.45, p< 0.001), with C. parapsilosis showing significantly better survival than all other species (p< 0.01 for all pairwise comparisons). Median survival times varied markedly: C. tropicalis (26.0 days), C. albicans (38.1 days), C. glabrata (53.0 days), while C. parapsilosis median survival exceeded 100 days. Age emerged as a critical prognostic factor across all species, with mortality exceeding 70% in patients ≥80 years regardless of causative species. The mortality gradient was particularly steep for C. tropicalis, rising from 57.1% in patients 0-19 years to 85.7% in those ≥80 years. No significant gender-based survival differences were observed.

**Conclusion:**

This comprehensive survival analysis demonstrates significant species-specific differences in candidemia outcomes. C. parapsilosis infections have substantially better prognosis compared to other species, particularly C. tropicalis which shows the poorest outcomes. Advanced age represents a critical prognostic factor independent of species. These findings emphasize the importance of rapid species identification for patient risk stratification and suggest that different Candida species may require tailored therapeutic approaches to improve outcomes.

**Disclosures:**

All Authors: No reported disclosures

